# Transcriptional responses in Honey Bee larvae infected with chalkbrood fungus

**DOI:** 10.1186/1471-2164-11-391

**Published:** 2010-06-21

**Authors:** Katherine A Aronstein, Keith D Murray, Eduardo Saldivar

**Affiliations:** 1 Honey Bee Research Unit, USDA-ARS, Weslaco, TX 78596, USA

## Abstract

**Background:**

Diseases and other stress factors working synergistically weaken honey bee health and may play a major role in the losses of bee populations in recent years. Among a large number of bee diseases, chalkbrood has been on the rise. We present here the experimental identification of honey bee genes that are differentially expressed in response to infection of honey bee larvae with the chalkbrood fungus, *Ascosphaera apis*.

**Results:**

We used cDNA-AFLP ^®^Technology to profile transcripts in infected and uninfected bee larvae. From 64 primer combinations, over 7,400 transcriptionally-derived fragments were obtained A total of 98 reproducible polymorphic cDNA-AFLP fragments were excised and sequenced, followed by quantitative real-time RT-PCR (qRT-PCR) analysis of these and additional samples.

We have identified a number of differentially-regulated transcripts that are implicated in general mechanisms of stress adaptation, including energy metabolism and protein transport. One of the most interesting differentially-regulated transcripts is for a chitinase-like enzyme that may be linked to anti-fungal activities in the honey bee larvae, similarly to gut and fat-body specific chitinases found in mosquitoes and the red flour beetle. Surprisingly, we did not find many components of the well-characterized NF-κB intracellular signaling pathways to be differentially-regulated using the cDNA-AFLP approach. Therefore, utilizing qRT-PCR, we probed some of the immune related genes to determine whether the lack of up-regulation of their transcripts in our analysis can be attributed to lack of immune activation or to limitations of the cDNA-AFLP approach.

**Conclusions:**

Using a combination of cDNA-AFLP and qRT-PCR analyses, we were able to determine several key transcriptional events that constitute the overall effort in the honey bee larvae to fight natural fungal infection. Honey bee transcripts identified in this study are involved in critical functions related to transcriptional regulation, apoptotic degradation of ubiquitinated proteins, nutritional regulation, and RNA processing. We found that immune regulation of the anti-fungal responses in honey bee involves highly coordinated activation of both NF-κB signaling pathways, leading to production of anti-microbial peptides. Significantly, activation of immune responses in the infected bee larvae was associated with down-regulation of major storage proteins, leading to depletion of nutritional resources.

## Background

The health of managed honey bees is often compromised by a variety of factors including nutritional stress, medications, parasites, and diseases. One such disease is chalkbrood, [1,2], caused by the fungus, *Ascosphaera apis *(Maassen ex Claussen) [3,4]. Although it does not normally kill an entire bee colony, the fungus causes significant mortality of brood, leading to gradual deterioration of the colonies. Since there are still no chemicals approved for control of chalkbrood [1], improved genetic stocks and good management are the most preferred tactics against this disease. Knowledge of molecular mechanisms controlling honey bee immune responses to pathogens would be expected to enhance our capability to further improve genetic stocks.

Recently, most molecular components of the honey bee humoral immune defenses were described by Evans et al. [5], and nearly all of them have dipteran homologues with conserved functions. For the first time, Evans et al. [5] systematically described members of the two principal NF-κB/Rel immune signaling pathways, Toll and the Immune Deficiency (IMD) pathways, providing a unique opportunity for further investigations of host-pathogen interactions in studies of honey bee diseases. As a result, it is now possible to link host innate immune responses to specific diseases.

In this study we attempted to elucidate dynamic changes in bees' transcriptional responses to mycosis using an in vitro bioassay and the natural mode of brood infection with *A. apis *spores. This research is built on the previous genomic and molecular insect studies that demonstrated that combined cellular and humoral immune responses to various microbes involve a large variety of processes, including proliferation of hemocytes, and activation of proteolytic and immune signaling cascades [6-13]. Phagocytosis and encapsulation are the most common insect anti-fungal defenses [12-17]. In addition, current molecular models suggest that humoral immunity play very important role in arthropods' antifungal defenses and mostly controlled by Toll signaling, leading to production of the antimicrobial peptides (AMPs) [6,7,12,18-20]. The existence of a large number of Toll-like receptors in insect genomes (e.g., 9 in *D. melanogaster*, 11 in *A. gambiae*, and 5 in *A. mellifera*) [5] may be due to redundancy, differential roles in insect development, or other specialized functions. New evidence suggests that some of the AMPs can be activated synergistically by the Toll and IMD-Rel pathways, indicating their cross-regulation [10,11,21]. In addition, some evidence indicates that the production of the AMPs can also be mediated by the apoptosis-associated c-Jun N-terminal kinases (JNK) branch of the IMD pathway (IMD-JNK) [22,23]. This apparent redundancy in immune signaling may provide an additional level of protection against microbial pathogens.

In an effort to identify essential humoral components involved in the honey bee's response to mycosis, we utilize amplified fragment length polymorphism (cDNA-AFLP) technology followed by quantitative real-time PCR (qRT-PCR) to monitor quantitative changes in the expression profile of transcripts. cDNA-AFLP is one of a few methods capable of screening for differentially expressed transcripts (up- as well as down-regulated) and finding genes that have not yet been previously identified or predicted from sequence analysis. However, it should be noted that molecular components that are controlled by posttranscriptional regulation or by posttranslational modification are expected to escape this analysis. Here we have also probed an additional set of previously identified immune-related genes using qRT-PCR to better understand the role of NF-κB signaling and changes in the level of transcripts during progression of the disease.

## Methods

### Infection Bioassay

One-day old worker bee larvae (mean weight 0.72 mg) were collected for immune challenge assay in a FALCON^® ^6-Well Non-Tissue Culture treated plate as described by Aronstein and Saldivar, 2005 [24]. One black chalkbrood mummy was crushed in 1.5 ml of mixed larval diet [25] and fed to the experimental groups of larvae (n = 90) at a final concentration of 1×10^5 ^spores per larva. A small sub-sample of fungus was analyzed using the PCR approach as described by Murray et al. [26] to confer fungal species. Thirty larvae were placed in each well containing 250 μl of the spore-containing diet. Subsequent feedings were done as needed with pure diet only. Control larvae (n = 90) were treated the same way with the exception that no feedings contained fungal spores. Culture plates were incubated at 95% RH and 33°C as described by Aronstein et al. [27]. Samples of ten larvae were collected from each group (infected and control) at 24 and 36 h time points. Larvae were preserved in RNA*later*^® ^(Ambion, Austin TX) and stored at -20°C until further analysis. The bioassay was repeated twice, and samples were analyzed separately by cDNA-AFLP.

### RNA isolation and cDNA synthesis

Total RNA was isolated from each sample (pool of ten larvae) using an RNeasy^® ^Mini Kit (Qiagen, Valencia CA) following the manufacturer's protocol, which included the removal of DNA from the samples using the gDNA Eliminator spin columns. To test for residual DNA contamination, 1 μl of the total RNA was used as template in PCR, using primers for the defensin gene fragment (Table 1), which contains a 286 bp intron. Samples that tested positive for the presence of the intron were treated with Ambion's DNA-*free*™ Kit until DNA was not detectable. Isolation of messenger RNA from the total RNA was done using MicroPoly(A)Purist™ (Ambion) following the manufacturer's protocol. A 1.0% denaturing agarose/formaldehyde gel was used to test the integrity and quantity of the RNA. Quantification was verified using an Eppendorf BioPhotometer with samples diluted in TE (10 mM Tris-HCl pH 8.0, 1 mM EDTA) and incubated for 10 min at 60°C. The poly-A RNA (500 ng) was used in the synthesis of double-stranded cDNA using SuperScript™ Double-Stranded cDNA Synthesis Kit (Invitrogen Life Technologies, Carlsbad CA), and oligo-dT primers according to the manufacturer's protocol.

**Table 1:** List of primers and annealing temperatures (Tm)°C used in qRT-PCR, and NCBI accession numbers

Gene and GenBank number	Tm°C	Forward Primers 5'-3'	Reverse Primers 5'-3'
Abaecin [U15954]	65°C	GGTAGTGATATTTATCTTCGC	TTGAGGCCATTTAATTTTCGG

Defensin-1 [U15955]	65°C	GTTGAGGATGAATTCGAGCC	TTAACCGAAACGTTTGTCCC

Lysozyme-1 [XM_001120995]	67°C	GGAGGCGAGGATTCTGACTCAATG	TGTTGCATATCCCTCCGCTGTG

Hexamerin 70b [XP_392868]	67°C	CCGCTCTTCAAATGTGGTCTAC	GATAGGTAAAAGGTTTGTGGTTC

Vitellogenin [XM_392349]	65°C	TTCTGATAAAGGCGTTGCTCAC	CTCGTCGTCGGTCGGAACTT

Trypsin like Serine protease [XM_393882]	65°C	TTGTTTACCGGCGAAAAATC	ATGTTCACGACCACATCCAA

Glycosyl hydrolase 18 [XM_397146.3]	67°C	GTGGTGGCAAACAAGCTGAT	CGCTGCAAAATTGTTCCACGA

MyD88 [XM_396644]	65°C	GAGAGGTCTTGCTCATTTATGC	TCTCAAGTTTATCCACCATTTCA

Actin [AB023025]	67°C	GAAATGGCAACTGCTGCATC	GAGATCCACATCTGTTGGAA

### Gene expression analysis

**Figure 1:**
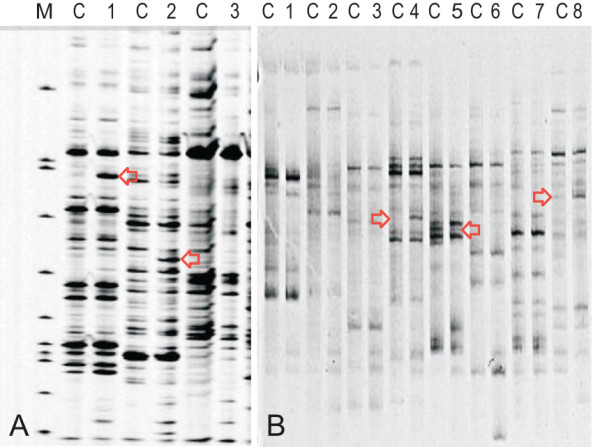
**Polyacrylamide gel electrophoresis**. cDNA-AFLP PCR products generated using different selective (2^+^/2^+^) primer combinations for the 24 h time point. PCR fragments that are in the range of 100-400 bp have been visualized using two different types of the polyacrylamide gels (A) PCR fragments were separated using 6.5% polyacrylamide gel and NEN Model 4300 DNA Analyzer (B) fragments separated on 8% polyacrylamide that ran on a V16-2 gel system apparatus and stained with 10,000× dilution of SybrGold for 20 minutes. Lanes: (M) DNA marker; (C) control group; (1-8) denotes treated group plus the primer combination used to generate the PCR product. Each pair of a control sample and an experimental sample (e.g., C1, C2, C3) was generated by PCR amplification using different primer sets (1-8). Some of the differentially expressed PCR fragments are indicated by arrows.

Global gene expression profiles of infected vs. uninfected bee larvae were determined using a cDNA-AFLP approach. To limit the number of reactions and subsequent gel runs to a manageable number, only the 24 h time point samples were used in the cDNA-AFLP analysis. Double-stranded cDNA samples were used as templates for AFLP using an AFLP^® ^Expression Analysis Kit (LI- COR Biosciences, Lincoln NE) with a slightly modified version of the manufacturer's protocol. Briefly, ~250 ng of double stranded cDNA samples were digested with *Taq*I and *Mse*I restriction enzymes and *Taq*I/*Mse*I adapters were ligated to the fragments. A 10-fold dilution of the ligation reaction was used in a pre-amplification reaction with non-selective primers complementary to the adapter sequences to enrich the templates. This was followed by the selective amplification of the resulting PCR products with sixty-four primer sets *Taq*I (T+2)/*Mse*I (M+2) containing 2 selective nucleotides at the 3' end of each primer. That is, each of the eight T+2 primers (T-AG, T-AC, T-CA, T-CT, T-GA, T-GT, T-TC, T-TG) were used with eight different combinations of M+2 primers. By multiplying the number of unique selective PCR reaction samples by the number of distinguishable fragments seen from a single sample, we estimate that a total of 7400 transcriptionally-derived fragments were detected. We tested two different types of gels to resolve PCR fragments (Fig. 1A-B): 6.5% polyacrylamide gels run on the NEN Model 4300 DNA Analyzer system (LI-COR BioScience, Lincoln NE) which automatically generates a gel image, and 8% polyacrylamide gels (SequaGel Kit, National Diagnostics, Atlanta GA) run on a V16-2 gel system apparatus (Labrepco, Horsham PA). The 8% gels were stained with SybrGold (Invitrogen Life Technologies, Carlsbad CA). Bands were visualized on the GEL DOC XR system and the Quantity One software Version 4.6 (Bio-Rad, Hercules CA). We ultimately chose to use the 8% gels due to the ease of excising the bands from the gel as opposed to the tedious and time consuming process of recovering PCR products from a gel run on the NEN Model 4300 DNA Analyzer system. Bands that showed clear differences in intensity were excised and eluted from gels after incubating the gel plugs overnight in water. The differentially expressed transcripts were then re-amplified using the non-selective primers. The resulting products were used as template for a second round of amplification with the selective primers. The PCR amplification products were gel purified (Qiagen, Valencia CA), cloned into the pCR^® ^2.1 vector of the TOPO-TA cloning kit (Invitrogen Life Technologies, Carlsbad CA), and sequenced (SeqWright Inc., Houston TX). For each excised fragment, multiple clones were sequenced in order to assess the purity and thereby avoid analysis of uninteresting background fragments.

### Bioinformatics

Candidate genes were compared to sequences in GenBank http://www.ncbi.nlm.nih.gov. tblastx and blastx searches were done against translated nucleotide databases and the non-redundant protein databases respectively. We have also determined the location of each candidate gene on the *A. mellifera *genome linkage groups using BeeBase dataset http://www.beebase.org/. The deduced amino acid sequences were analyzed using NCBI Conserved Domain Database (CCD) search service version v2.14 and NCBI Conserved Domain Architecture Retrieval (CDART) tool.

### Quantitative gene expression analyses

Differentially expressed genes identified via cDNA-AFLP as well as other genes expected to be involved in the insect's immune responses were analyzed using qRT-PCR. A list of genes, primers, and annealing temperatures used in this study to examine the level of gene expression is provided in Table 1. The honey bee actin gene was selected as a reference gene to normalize qRT-PCR experiments since it was validated among the most stably expressed genes tested in various honey bee tissues and developmental stages [28,29]. Furthermore, we validated the stability of actin expression during early larval development before using it in this study. The qRT-PCR reaction mix (20 μl) consisted of 1.0 U of GoTaq^® ^Flexi DNA polymerase (Promega Co., Madison, WI) with the colorless 5× GoTaq^® ^Flexi buffer, 0.25 mM dNTP mix, 2.5 mM MgCl_2_, 0.3 μM of each primer, 0.75 μl of a 1/1000 stock dilution of Sybr-Green (Invitrogen Corp.), and 1 μl of cDNA. Reactions ran for 40 cycles at 95°C (20 s), 60°C or 65°C (30 s) depending on the gene (Table 1), and 72°C (30 s) after initial denaturing at 95°C for 3 minutes. Negative control reactions were included in each run and contained all reaction components except the template. All reactions were done in triplicate. Fluorescence was measured at the end of the elongation stage of every PCR step. To quantitate gene expression, standard curves were amplified simultaneously with the experimental samples using a serial dilution of known amount of column purified (Qiagen, Valencia CA) gene-specific DNA template. The quantity (ng/μl) of the DNA template for the standard curves was determined with Eppendorf's BioPhotometer and validated by running the sample on an agarose gel prior to use. The templates for the standard curves were then diluted to either picograms (pg) or femtograms (fg) to ensure that the experimental samples amplified within the range of the standard curve. Furthermore, a melting curve analysis was performed subsequent to each qRT-PCR run to confirm specificity of the amplified product.

### Data analysis

For each qRT-PCR run, the baseline was set to the point at which fluorescence was 10 times greater than the standard deviation of mean fluorescence of the cycle range from cycle one to cycle five. The standard deviation and average were calculated for each qRT-PCR run. The abundance of nucleic acid in each sample was normalized by dividing the calculated abundance of the gene of interest by the abundance of actin. Data was analyzed with the GraphPad Instat (GraphPad software, San Diego, CA) using a one-way ANOVA and Tukey's post-test with *P *< 0.05 being considered statistically significant.

## Results and Discussion

### Analysis of gene transcripts according to functional classes

Within 24-hr post infection, honey bee larvae in the experimental group significantly reduced their rate of feeding, became visibly sluggish at 36 h, and at 48 h post-infection showed no signs of life. In contrast, 80 % larvae in control survived up to 10 days post infection. Samples of ten larvae were collected from experimental and control groups at 24 and 36 h post-infection. cDNA was made, followed by AFLP analysis as described above. At 24 h post-infection, a small fraction of transcript fragments differed in intensity between experimental and control samples. A total of 98 reproducible polymorphic cDNA-AFLP fragments were excised and sequenced.

The transcriptional profiles of the three genes were then verified by qRT-PCR at 24 h and 36 h post-infection. In addition, five other gene transcripts were selected for the qRT-PCR analysis based on their functional classes. The corresponding entire coding regions of the newly identified transcripts were determined using NCBI tblastx and blastx database searches followed by CDD analysis (Table 2). To minimize the false positives, we used only sequences with an E-value of 0.006 or less for inclusion in the follow-up analysis. The identification of functionally characterized domains in putative protein sequences provided the first indication of their molecular and cellular functions. Among the identified genes differentially expressed in chalkbrood-infected bee larvae were those sharing sequence similarities with many known insect genes previously implicated in stress-related cellular processes. As seen in Table 2, identified transcripts encode proteins potentially involved in vesicular transport (Sec61), protein ubiquitination (26S), RNA processing and transcriptional regulation (L23, SAM-MT), detoxification of xenobiotics by the microsomal cytochrome P450s (cyb5), multi-drug transport (ABC1), nutritional and hypoxic stress responses (Hex 70b, PFKFB), immune signaling (Lmtk3, Tryp_SP), and antifungal activity (Glyco_18). Additional file 1TableS1 contains a complete list of cloned honey bee transcripts.

**Table 2:** Cloned transcripts after BLAST search with domain and predicted function

Gene Name	GenBank Accession Number	Functional domain	Predicted Protein Function
↑ similar to Lemur Tyrosine Kinase 3 (PTK)	XM_624449	catalytic PTKc_Aatyk, cd05042	signalling/Apoptosis-associated tyrosine kinase (Aatyk)

**↑ **similar to CG8172-PA, variant 1	XM_393882	Tryp_SPc, cd00190	trypsin like serine protease

↑AmelNPC2-like	XM_001120140	NPC_2-like	lipid binding protein

↑Hexamerin 70b	XP_392868	Hemocyanin_M; Hem_C; Hem_N.	larval storage protein

↓26S proteasome non-ATPase regulatory subunit 9	XM_623256.2	PDZ	degradation of ubiquitinated proteins and apoptotic cells.

**↑ **Glycosyl hydrolase 18 -like	XM_397146.3	Glyco_18	glycosyl hydrolase/chitinase-like protein

**↑**ATP-binding cassette (ABC1) proteins	XM_394305.3	ABC_subfamily_A, d03263.	ABC-type multi-drug transport system

**↑ **(SAM-MT)-like 6	XM_623529	pfam08242, Methyltransf_12	transcriptional regulation

↑Sec61 beta subunit	XM_001119885		protein transport, hypoxic respond to stress

↑ Ribosomal protein L23	XM_392812.3		Ribosome biogenesis, rRNA processing

↑similar to CG17838-PE, isoform E	XM_392307	RNA recognition motif (RRM)	mRNA processing, biogenesis

↑6-phosphofructo-2-kinase/fructose-2,6-biphosphatase 1(PFKFB)	XM_393453.3		energy metabolism, hypoxic response

**↓**Osiris 6 (Osi6)	XM_001121541.1	(DUF1676)	transmembrane proteins of unknown function

↓Cytochrome b5-related (*cyb5*)	XM_001120985.1		response to oxidative stress

Based on the CDD prediction analysis, we selected for qRT-PCR analysis several of the differentially-regulated genes (e.g., *AmelCht*, *sp6*, *hex 70b*) known to be important for response to stress and/or immune defenses in other insects.

**Figure 2:**
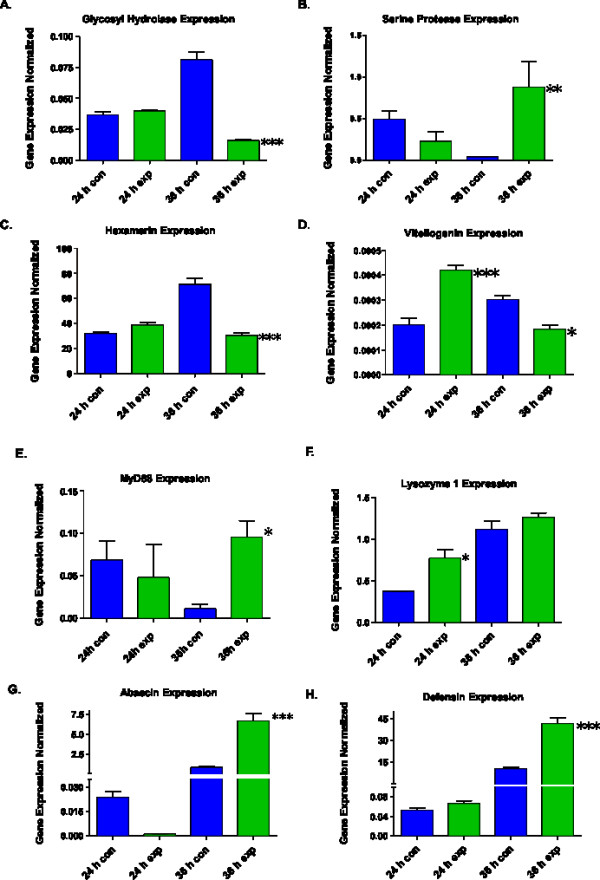
**qRT-PCR analysis of selected cDNA-AFLP transcripts (A-C) and transcripts not derived from cDNA-AFLP (D-H):**. (A) Glycosyl Hydrolase, (B) Serine Protease, (C-D) storage proteins *Hex 70b *and *Vg*, and (E-H) components on the honey bee immune response pathways including anti-microbial peptides. Control group of samples are represented by blue bars, the experimental samples are represented by green bars. Samples were done in triplicate and normalized by dividing the amount (pg or fg as described in Materials and Methods) of the gene of interest by the amount for the housekeeping gene, actin. Results are reported as an average of the triplicates plus the standard deviation. Significant by ANOVA at P < 0.05(*), P < 0.01(**), and P < 0.001(***).

The cDNA-AFLP method surprisingly did not detect any components of the well characterized Toll and IMD signaling pathways. However, we decided to analyze components of the Toll pathway using qRT-PCR, since this pathway is activated in response to fungal infection in *Drosophila *[12]. Because some of the transcripts were determined to be differentially expressed using qRT-PCR, it is clear that a number of differentially-regulated transcripts were missed from the cDNA-AFLP analysis, probably due to short-comings of the method itself. The short-comings of the cDNA-AFLP method are also evident from the discrepancies in the gene expression profiles determined by these two methods. For example, the *AmelCht *transcript was up-regulated 24 h post-infection if analyzed via cDNA-AFLP, but no significant up-regulation of this gene was determined by qRT-PCR at the same time point (Fig. 2A). Of course, any pathways activated by post-transcriptional mechanisms would not be detected by cDNA-AFLP.

### Apoptosis-associated phosphorylation

One of the up-regulated genes in infected larvae encodes a protein similar to apoptosis-associated lemur tyrosine kinase 3 *(Lmtk3) *[GenBank:XM_624440.2]. This protein includes an apoptosis-associated tyrosine kinase catalytic domain (AATYK), which has high homology to the same domain in the mouse homologue [30]. The induction of the mouse AATYK in cerebellar granule cells induced apoptosis in these cells [31-33]. It is therefore conceivable that *A. mellifera Lmtk3 *is involved in apoptotic removal of damaged cells in diseased larvae. AATYK tyrosine kinases may also be involved in production of AMPs, since they were implicated in activation of the apoptosis-associated JNK signaling pathway in vertebrates [22,23].

### Degradation of proteins

Progressive accumulation of abnormal proteins is one of the most widely reported stress-related changes in eukaryotic cells [34,35] and under normal conditions it is precisely controlled by intracellular degradation. The ubiquitin-proteasome pathway is critical in this process. The proteins targeted for degradation are marked by covalent attachment of ubiquitin (Ub) and are degraded in an ATP-dependent manner by the 26S proteasome. In addition, the 26S proteasome mediates intracellular degradation to control levels of key regulatory proteins [34]. We found that a gene similar to the 26S proteasome non-ATPase regulatory subunit 9 [GenBank:XM_623256.2] was down-regulated in infected honey bee larvae. The down-regulation of the 26S proteasomal subunit would be expected to decrease proteasomal activity in mycosed bee larvae and result in a failure to cope with proteotoxic damage, similar to a disorder in *Drosophila *that affects proteasome activity [35]. In addition, ATP-depletion expected to occur during the demise of the larvae would likely interfere with the ATP-dependent processes of 26S proteasome assembly and normal protein turnover. Therefore, low ATP concentration may be part of a regulatory signal which inhibits transcription of this gene.

### Anti-fungal activity

We have identified a chitinase-like sequence (*AmelCht*) [GenBank:XM_397146] that encodes a putative glycosyl hydrolase 18. The *AmelCht *transcript was up-regulated 24 h post infection, however its abundance significantly dropped (about 6.8-fold) by 36 h post infection (q = 18.94; P < 0.001) (Fig. 2A). Using the NCBI CDART tool, we found that *AmelCht *contains one catalytic (Glyco_18) and one chitin binding Peritrophin-A (CBM_14) domain. Members of this group have been characterized as gut- and fat body-specific chitinases [36,37], which are major sites of immune activity in insects. The NCBI BLAST search revealed that the *AmelCht *gene has high similarity with *Locusta migratoria manilensis *midgut chitinase [GenBank:EF090723], *Anopheles gambiae *gut-specific chitinase (AgChi-1) [GebBank:AF008575], and *Tribolium castaneum *chitinase 8 (*Cht8) *[GenBank:XM_967543] each of which also contains one catalytic and one chitin binding domain [36]. Glyco_18 chitinases previously identified in animals and plants have been implicated in anti-fungal activity [38,39], including reductions in hyphal diameter, hyphal branching and conidia size [40]. The decrease in *AmelCht *transcript 36 h post-infection (Fig. 2A) could be a result of damage to the larvae's gut tissues as a result of mycosis, which would be expected to impair the ability of bee larvae to digest food.

### Immune signaling

Trypsin-like serine protease (SP) transcript variant 1 [Genbank:XM_393882] was significantly (24.1-fold) up-regulated in infected larvae at 36 h post-infection (q = 8.52; P < 0.01) (Fig. 2 B). Predicted amino acid sequence analysis revealed that this honey bee SP contains one catalytic (Tryp_SPc) domain with trypsin specificity, and one CLIP 1_a _domain containing the DVAL catalytic site. According to Zou et al. [41], this honey bee SP [GI:CG8172], which is classified as *Amel *cSP6 trypsin-like serine protease, was transcriptionally up-regulated in response to septic injections of adult workers with Gram-positive bacterial spores. In arthropods, CLIP-domain SPs mediate innate immunity and embryonic development [42,43]. Although function prediction of insect SPs is a very challenging task, the co-existence of these two functional domains suggests synergistic function for them in immune responses [7,44,45].

### qRT-PCR analysis of previously known immune-related transcripts not derived from cDNA-AFLP

Although our cDNA-AFLP method did not detect transcriptional changes in genes of the two primary immune signaling cascades, the Toll and IMD pathways, we decided to probe some of the Toll members using qRT-PCR since the Toll pathway is typically activated by fungi [12]. Surprisingly, none of the five honey bee Toll receptors had any significant change in the expression level (not shown). Considering that invertebrate Toll signaling is strictly MyD88-dependent [46,47,11], we decided to investigate the expression profile of this universal Toll adaptor in infected bee larvae. In response to infection, the level of the MyD88 transcript had no significant changes at 24 h, but was up-regulated 8.3-fold over the control at 36 h post-infection (q = 5.9, P < 0.05) (Fig. 2E). The fact that neither the Toll receptor genes nor MyD88 were detected by the cDNA-AFLP method as being differentially-regulated at 24 h post-infection was therefore confirmed by the qRT-PCR results.

In agreement with our previous study [24], we observed that the AMP genes, abaecin and defensin-1 (Table 1), were responsive to stress induced by mycosis. Each was significantly up-regulated at 36 h post infection (q = 21.21; P < 0.001 and q = 15.81; P < 0.001) respectively (Fig. 2G, H). In the experimental groups of larvae we recorded an 8.1-fold increase in the transcript level for abaecin and a 4.0-fold increase for defensin. The immune-inducibility of AMPs is a very important indicator of NF-κB signaling, [6,21,22,48,49].

In addition to their well known antibacterial activity, lysozymes have been implicated in antifungal and antiviral activity [50]. Honey bee C-type lysozyme-1 (Table 1) showed a 2.1-fold increase at 24 h post infection (q = 5.2; P < 0.05) (Fig. 2F). An increased level of lysozyme may improve the ability of phago-lysosomes to break down fungal mycelia and thereby provide an additional level of antifungal defense to infected larvae.

### Lipid binding

The cDNA-AFLP analysis revealed a significant up-regulation of a honey bee *npc2*-like transcript that encodes a secreted lipid-binding protein. *Drosophila npc2*-like genes play roles in regulating sterol homeostasis and 20-hydroxyecdysone (20E) biosynthesis [51,52]. The *npc2 *gene transcript has been associated with the regulation of honey bee worker sterility [53].

In vertebrates, NPC2 has been associated with a type of lipid-storage disorder called Niemann-Pick type C2 disease [54]. Similarly to mammalian NPC disease, the *npc2 *mutation in *Drosophila *was linked to apoptotic neurodegeneration [52]. Furthermore, vertebrate NPC2 has a similar binding fold as MD-2 (a lipopolysaccharide-binding protein), which is indispensable for toll-like (TLR4) receptor signaling in the vertebrate immune system [55]. Although the extracellular events proceeding the activation of Toll signaling are still poorly understood [12,56], the up-regulation of a lipid-binding protein which may be involved in activation of toll signaling in fungus-challenged bee larvae is intriguing.

### Multi-drug transport

An ATP-binding cassette (ABC1) member1 gene [GenBank:XM_394305.3] was up-regulated in infected honey bee larvae. Such ATP-binding proteins belong to the ABC transporter superfamily that is involved in transport of a variety of macromolecules across biological membranes. In addition, members are known to play a prominent role in the absorption, distribution, metabolism, and excretion of xenobiotics, and therefore members of this family have been implicated to play significant role in response to diseases [57].

### Genes with putative roles in responses to various stressors

Similar to findings reported by Zhou et al. [58] we have found a number of differentially expressed *A. mellifera *genes (Table 2) potentially involved in transcriptional regulation, protein secretion and translocation, ubiquitination, and energy metabolism.

The S-adenosylmethionine dependent methyltransferase-like 6 (SAM-TM) [GenBank:XM_623529] was up-regulated more then 5-fold in infected bee larvae. It encodes an enzyme with a methyltransf_12 domain (pfam08242) that catalyzes the methylation of proteins, nucleic acids or small molecules and may be involved in transcriptional regulation, RNA processing and signal transduction [59], and have been implicated in disease responses [60].

The *Sec61 *transcript [GenBank:XM_001119885] was up-regulated approximately 2-fold. This gene encodes a component of the endoplasmic reticulum (ER) protein translocation apparatus. It was demonstrated previously in yeast that high levels of expression of Sec61 homologue suppressed components of the exocyst protein secretory system [61]. Exocyst complex is a critical mechanism contributing to the formation and maintenance of cellular membranes [62-64]. In Drosophila, mutations in several Sec subunits affected synthesis of membrane proteins, blocked their transport to the cell surface, resulting in hypoxic cell death [62,63,65].

The ribosomal protein *L23 *[GenBank:XM_392812.3] transcript was up-regulated approximately 2- fold in infected honey bee larvae. This is a multi-functional protein essential in early ribosome assembly events as well as in early and late stages of rRNA processing and biogenesis [66]. This protein interacts with several protein folding and translocation components, including the Sec61 subunit mentioned above.

The up-regulated nuclear ribonucleoprotein isoform E [GenBank:XM_392307] gene is similar to *Drosophila *and *Aedes aegypti *hnRNPs. hnRNPs encode RNA binding proteins that play fundamental roles in the post-transcriptional control of gene expression, localization and translational regulation of mRNAs [67].

A gene similar to Cytochrome b5 (cyb5) [GenBank:XM_001120985.1] was significantly down-regulated (> 10-fold) in the infected honey bee larvae. This gene encodes a cyb5-related hemoprotein and has been implicated in oxidation of endogenous and xenobiotic substances by the microsomal cytochromes (CYP) P450s [68]. It has been previously reported that infection inhibits the expression of cytochrome P450 detoxification genes in vertebrates [7]. This result is consistent with the association of immune defense activation and the repression of oxidation by the P450s.

Another transcript, 6-phosphofructo-2-kinase (PFKFB) [GenBank:XM_393453.3] encoding a protein similar to human liver isozyme Fru-2,6-P2ASE was significantly up-regulated (> 10-fold) in mycosed larvae. In eukaryotes, this enzyme is involved in regulation of glucose metabolism. The up-regulation of this glycolytic enzyme in diseased animals is a hallmark of the hypoxic response to reduced oxygen conditions [69,70].

### Nutritional stress response

Since overall health, life span, and the level of oxidative stress of bees depend on the level of the major storage proteins, hexamerins (*Hex) *and vitellogenin (Vg) [71], we measured the expression levels of *Hex70b *and *Vg *in infected and healthy larvae (Fig. 2D). Using cDNA-AFLP, we determined that the hexamerin (*Hex 70b) *gene [GenBank:AY601637] was up-regulated 24 h post infection, followed by a significant decrease in gene expression at 36 h determined by qRT-PCR (q = 13.8; P < 0.001) (Fig. 2C). Insects store a large amount of hexamerins during larval stages. These are large multifunctional storage proteins composed of several subunits. They are synthesized by larval fat body cells and then released into the hemolymph [72]. Four *A. mellifera *hexamerin subunits (HEX 70a, HEX 70b, HEX 70c, and HEX 110) were previously described in the developing honey bee [73], and some of them (Hex70a, Hex 70b and Hex 110) have been functionally characterized [73-75].

Recent research has shown that the hexamerins may play a far more complex role in insect biology and development then just a source of amino acids for tissue reconstruction. They may serve as an environmentally and nutritionally responsive mechanism helping animals to survive nutritional, oxidative and disease stressors [71,76,77]. Current research suggests that levels of storage protein expression rely heavily on nutritional status and are in direct correlation with the overall health status of larvae [78]. Furthermore, it has been suggested that there is a trade-off between immune stimulation and expression of storage protein genes [79]. Therefore, changes in storage protein expression may reflect altered energy metabolism in starving larvae that in our study had reduced feeding by 24 h and were in a lethargic state by 36 h post infection. Consistent with results by Guidugli et al. [80], expression of the other major storage protein, Vg [GenBank: XM_392349], was determined in bee larvae in this study using previously known gene sequence (Table 1) and qRT-PCR. Similar to *Hex 70b*, the expression level of *Vg *was affected by fungal pathogenesis. First it showed a significant increase in expression at the 24 h time point (q = 10.40; P < 0.001). As the disease progressed, that was followed by a significant decrease in *Vg *transcript abundance (q = 5.67; P < 0.05) (Fig. 2D).

### Proteins of unknown function

An *A. mellifera *transcript similar to Osiris 6 [GenBank:XM-001121541] was down-regulated in challenged larvae. It belongs to a novel gene family, predicted as type I transmembrane proteins (DUF1676) of unknown function, possibly with a role in signaling [81]. *A. mellifera *Osiris 6 showed high homology to sequences found in other insects: 62% identity with *Nasonia vitripennis *[GenBank:XM_001602335], 37% with *Culex pipiens quinquefasciatus *[GenBank:XM_001849645.1], and 41 % with *Drosophila melanogaster Osiris 6 *[GenBank:NM_141368.2]. We found a cluster of 17 *Osiris *genes on AmeLG15 (NW_001253127.1) similar to a cluster of 20 genes (*Osiris 1 *- *23*) in the Triplo-lethal region (Tpl) of the *D. melanogaster *genome [81]. *Osiris *genes are also known in other insects (e.g., *Bombyx mori*, *Cicindela campestris*, *Toxoptera citricida*), but no homologues have been found in any other phyla. That led to speculation that the function of the Osiris proteins may be insect or Arthropod specific [81].

Several other sequences identified in this study had no apparent open reading frames. They were localized in very close proximity to annotated *A. mellifera *genes. We speculate that they could represent 5' regulatory elements or 3' untranslated regions of flanking genes. One down-regulated transcript was localized 230 bp upstream of transcription factor-like 4 [GenBank:XM_395909], a DNA-binding protein. Family members encode central regulators of mammalian global gene expression, cell proliferation and apoptosis. Another up-regulated transcript was localized at AmeLG6 [GenBank:NW_001253446.1], 199 bp downstream of similar to Cut Homeobox transcription factor [GenBank:XM_623854]. Cut proteins function as transcriptional repressors of genes specifying terminal differentiation in multiple cell lineages. A transcript [GenBank:XP_001121248] located 1961 bp downstream of *Tis11 *[GenBank:XP_001121248] was down-regulated in infected larvae. In mouse, TIS11 is recruited to heat shock-induced stress granules and known to appear in the cytoplasm of cells in response to various stressors.

## Conclusions

Using a combination of cDNA-AFLP and qRT-PCR analyses, we were able to determine several key events that constitute the overall effort in honey bee larvae to fight natural fungal infection.

Among them, activation of immune signaling associated with down regulation of major storage proteins, and activation of cellular machinery dealing with general stress responses and progressive accumulation of abnormal proteins. We showed here that chalkbrood infection had a global effect on several physiological pathways in bee larvae and caused dramatic changes in the regulation of a number of genes. Some of these transcripts could be disease-specific and some of them could play a more general role in survival of the mycosed larvae in response to nutritional deprivation and stress in general.

### Intracellular immune signaling

Despite extensive genomic and functional insect studies, there is still very little known regarding antifungal humoral immune defenses and specifically Toll signaling in the honey bee [5,24]. Although many components of Toll pathway are up-regulated in other insects in response to fungal infection, we did not find up-regulation of Toll genes in this study. However, the lack of Toll genes expression activation here may be due to the fact that Tolls are expected to be up-regulated in only a subset of tissues. Since we isolated RNA from the entire larvae, a transcription activation occurring in specific tissues only might have been masked. Nevertheless, based on the activation of MyD88 transcription in infected larvae, we suggest that *A. apis *does activate Toll signaling. MyD88 is a universal Toll adaptor protein, interacting with Tolls via Toll/IL-1R (TIR) domain [11,12,46,82,83]. Since Toll signaling in insects is strictly MyD88-dependent [11,46,47,82], MyD88 up-regulation implies activation of the Toll pathway. This provides the first evidence (albeit indirect) that fungal pathogenesis activates the Toll signaling pathway in the honey bee larvae.

In addition, the up-regulation of AMPs seen in this study is also consistent with activation of the Toll pathway. Granted, it is unknown which of the NF-κB pathways control expression of which AMP. However, a recent study by Schlüns and Crozier, 2007 [84] sheds some light on this question. By silencing IMD/Rel signaling, they showed that abaecin and hymenoptaecin (another AMP) expression is mostly regulated by the IMD pathway, whereas expression of defensin-1 was not effected in the *Rel^- ^*cells [84], indicating its control by Toll signaling. Thus, insects appear to rely upon the highly coordinated activation of both NF-κB signaling pathways [10,11,85] leading to production of multiple AMPs with distinct activities [21]. It is also interesting to note that while septic injection of fungal spores also up-regulates expression of all antimicrobial peptides [14,24], it is most likely induced by the mechanical injury.

### Stress responses

In addition to direct damage by the fungus itself during infection, bee larvae suffer physiological side effects due to activation of the host immune system [86]. Here we show that activation of immune-related transcripts is temporally associated with significant down-regulation of major storage proteins (e.g., *Hex *70b and *Vg*), which may serve as a source of amino acids for metabolism and development. This finding is consistent with recent report by Lourenco et al. [79], who showed a significant down-regulation of *Hex70a *and *Vg *in honey bees in response to bacterial infection.

Taken together, our findings showed that fungal infection had a profound effect on the overall health of bee larvae causing rapid decrease in the rate of larval feeding, and substantial decrease in transcription of storage proteins. In addition, competition for food between larvae and fungus further depleted larvae of the essential nutrients. Mycosis also affected expression of diverse families of genes, involved in critical functions related to transcriptional regulation, apoptotic degradation of ubiquitinated proteins, RNA processing, activation of immune signaling and up-regulation of AMPs' transcripts. Activation of multiple mechanisms in bee larvae dealing with fungal infection suggest that in addition to redundancy between different NF-κB signaling pathways, there is a crosstalk between immune and stress response mechanisms in insects. This combined effort of a wide variety of immune defenses and cellular stress response mechanisms are deployed to protect animals from damage inflicted by microbial pathogens. However, taking into account a high level of mortality of the infected larvae, it is apparent that insect innate immunity is no match for the impact of a massive mycosis. Clearly, behavioral adaptations in social insects provide more efficient mechanisms of protection against diseases at the colony level often at the cost of the individual colony members. Hopefully, development of new pathogen-specific pharmaceuticals and dietary supplements will assist honey bees in their struggle with this fungal pathogen.

While this study answers some questions, it brings up many more, and therefore provides the groundwork on which future studies of transcriptional and post-transcriptional responses of individual genes to fungal infection may build.

## Authors' contributions

ES conducted in vivo infection bioassays, carried out the data analysis, and contributed to manuscript. KDM participated in pathogen related work, interpretations of the results, and drafted the manuscript. KA conceived of the study, participated in its design and coordination, conducted the bioinformatics and wrote the initial version of the manuscript. All authors read and approved of the final manuscript.

## Supplementary Material

Additional file 1 **Table S1 - Complete list of cloned honey bee transcripts**. Cloned transcripts identified by cDNA-AFLP approach with the corresponding NCBI GenBank accession numbers and functional domains found in the predicted protein sequences. Arrows in front of the gene names indicate up- or down regulated transcripts in the experimental samples. Click here for file
