# Supplementary material for: Transcriptional responses in Honey Bee larvae infected with chalkbrood fungus
Source: BMC Genomics. 2010 Jun 21;11:391. doi: 10.1186/1471-2164-11-391 (PMC2996924; doi:10.1186/1471-2164-11-391)
Supplement: Additional file 1 — Table S1 - Complete list of cloned honey bee transcripts. Cloned transcripts identified by cDNA-AFLP approach with the corresponding NCBI GenBank accession numbers and functional domains found in the predicted protein sequences. Arrows in front of the gene names indicate up- or down regulated transcripts in the experimental samples. [file 1471-2164-11-391-S1.PDF]

**Gene Name**	**GenBank Accession Number**	**Functional domain**	**Predicted Protein Function**
↑ similar to Lemur Tyrosine Kinase 3 (PTK)	XM_624449	catalytic PTKc_Aatyk, cd05042	signalling/Apoptosis-associated tyrosine kinase (Aatyk)
↑ similar to CG8172-PA, variant 1	XM_393882	Tryp_SPc, cd00190	trypsin like serine protease
↑ AmelNPC2-like	XM_001120140	NPC_2-like	lipid binding protein
↑ Hexamerin 70b	XP_392868	Hemocyanin_M; Hem_C; Hem_N.	larval storage protein
↓ 26S proteasome non-ATPase regulatory subunit 9	XM_623256.2	PDZ	degradation of ubiquitinated proteins and apoptotic cells.
↑ Glycosyl hydrolase 18 -like	XM_397146.3	Glyco_18	glycosyl hydrolase/ chitinase-like protein
↑ ATP-binding cassette (ABC1) proteins	XM_394305.3	ABC_subfamily_A, d03263.	ABC-type multi-drug transport system
↑ (SAM-MT)-like 6	XM_623529	pfam08242, Methyltransf_12	transcriptional regulation
↑ Sec61 beta subunit	XM_001119885		protein transport, hypoxic respond to stress
↑ Ribosomal protein L23	XM_392812.3		Ribosome biogenesis, rRNA processing
↑ similar to CG17838-PE, isoform E	XM_392307	RNA recognition motif (RRM)	mRNA processing, biogenesis
↑ 6-phosphofructo-2-kinase/fructose-2,6-biphosphatase 1(PFKFB)	XM_393453.3		energy metabolism, hypoxic response
↓ Osiris 6 (Osi6)	XM_001121541.1	(DUF1676)	transmembrane proteins of

		unknown function
↓Cytochrome b5-related (*cyb5*)	XM_001120985.1	response to oxidative stress
↓AmeLGUn	NW001260288.1	230 bp up-stream of transcription factor-like 4 [XM_395909]
↑AmeLG6	NW_001253446.1	199 bp down stream of transcription factor similar to Cut [XM_623854]
↓ Group13.21	NW_001253078.1	1961 bp down stream of *Tiss 11,* [XP_001121248]
↑ Group3.7	NW_001253299	21960 bp up-stream of cacophony isoform A [XM_392298]
↑AmeLG7	NW_001253447	Osmotic avoidance abnormal protein 3 (Kinesin-like protein osm-3) [XM_395281]
↑Group14.21	NW_001253099	11554 bp up-stream of potassium channel [XM_392010]
↑Group7.16	NW_001253462	similar to CG4199-PD, isoform D [XM_625032]
↓GroupUn.132	NW_001260288	similar to CG11866-PA [XR_015106]
↑Group12.30	NW_001253057	similar to photoreceptor-specific nuclear receptor isoform b [XM_624039]
